# Current Update on PET/MRI in Gynecological Malignancies—A Review of the Literature

**DOI:** 10.3390/curroncol30010083

**Published:** 2023-01-12

**Authors:** Mayur Virarkar, Sai Swarupa Vulasala, Luis Calimano-Ramirez, Anmol Singh, Chandana Lall, Priya Bhosale

**Affiliations:** 1 Department of Diagnostic Radiology, University of Florida College of Medicine, 655 West 8th Street, C90, 2nd Floor, Clinical Center, Jacksonville, FL 32209, USA; 2 Department of Internal Medicine, East Carolina University Health Medical Center, 600 Moye Blvd., Greenville, NC 27834, USA; 3 Department of Diagnostic Radiology, The University of Texas MD Anderson Cancer Center, 1515 Holcombe Blvd., Houston, TX 77030, USA

**Keywords:** PET/MRI, gynecological malignancy, PET/CT

## Abstract

Early detection of gynecological malignancies is vital for patient management and prolonging the patient’s survival. Molecular imaging, such as positron emission tomography (PET)/computed tomography, has been increasingly utilized in gynecological malignancies. PET/magnetic resonance imaging (MRI) enables the assessment of gynecological malignancies by combining the metabolic information of PET with the anatomical and functional information from MRI. This article will review the updated applications of PET/MRI in gynecological malignancies.

## 1. Introduction

Gynecological malignancies are a common etiology of mortality and morbidity in women [1]. In 2022, they are estimated to be responsible for 115,130 new cases and 32,830 deaths in the United States [2]. Most of these cancers are staged using the International Federation of Gynecology and Obstetrics (FIGO) system based on clinical examination and diagnostic imaging, along with procedures such as colposcopy, cystoscopy, and rectosigmoidoscopy. For the past two decades, positron emission tomography (PET)/computed tomography (CT) has been the cornerstone for tumor staging, treatment, and recurrence. Magnetic resonance imaging (MRI) was evolving in parallel in evaluating tumor stage with its inherent soft tissue contrast, reduced radiation exposure, and high spatial resolution. MRI is employed in patients where radiation and tissue resolution are the major considerationsAt a later time, integrated PET/MRI gained popularity after 2011 and has become the research of interest. Since then, its clinical benefit has been well-studied in brain malignancies. However, it is still on the quest to confirm its importance in the rest of the cancers. MRI has the propensity to complement metabolic tumor evaluation obtained through PET imaging. The synchronous acquisition of anatomic layout on MRI and functional data on PET can enhance soft tissue assessment and characterization [3]. In addition, the tissue density on diffusion weighted imaging facilitates the local tumor invasion and distant metastatic spread. In this article, we elaborate on the basic technical outline, utility, advantages, and limitations of PET/MRI in gynecological malignancies.

## 2. Technical Background

A streamlined and competent workflow is necessary for the well-established recognition of PET/MRI in routine practice. The provider must be aware of the imaging modality and its lucrative benefits in specific disease populations. It must be followed by appropriate scheduling and proper communication between PET/MRI technologists and radiopharmacists. Once the imaging is scheduled, the pertinent protocol should be selected by the molecular imaging team to determine the radiotracer and by the radiology team to choose the body-site-specific MRI protocol (Table 1). Ultimately, close harmony between the interpreting physicians is essential to avoid conflicting image impressions between regional MRI and whole-body PET/MRI reports.

Over the past two decades, PET/CT has been the standard for evaluating oncologic conditions. In most institutions, the CT component is performed using a low-dose CT scan without contrast administration. However, such practice may result in poor tumor delineation, particularly in the case of pelvic malignancies. PET/MRI provides excellent tumor evaluation and metabolic information from the PET component. The currently available PET/MRI units have a 3-T magnet paired with PET detectors that can coexist and function with the MRI’s strong magnetic fields (Table 2). There are three major types of PET/MRI scanners: (i) Trimodal, (ii) Sequential, and (iii) Integrated systems. The trimodal system involves separate image acquisition through PET/CT and MRI scanners and software-based registration of acquired images. It allows the standard CT-based attenuation correction of the PET images. However, the limitations include potential misregistration due to patient transportation requirements and involuntary patient motion between the separate image acquisitions and the increased scanner space to accommodate the PET/CT and MRI. The sequential system can be described as the different PET and MRI acquisitions in the same room that contains a table that moves in a single track across the two scanners. Its advantage over the trimodal system is that it does not require the patient to be transported to an entirely different room.

The integrated system comprises solid PET-based detector rings placed inside the MRI gantry. Detector rings, such as semiconducting avalanche photodiodes, are smaller than traditional PET detectors and are compatible with strong MRI magnetic fields. While this configuration is still being studied, an alternate approach including silicon photomultiplier tubes has been reported. Compared with solid PET detectors, these tubes increase the detector response time, eliminate the PET and MRI circuity interference and enhance the scanner performance. Nonetheless, CT-based attenuation correction (CTAC) cannot be performed in sequential or integrated systems, thus posing a challenge to the engineers to optimize the MRI-based attenuation correction (MRAC). Initially, the T1-weighted Dixon MR sequences were used to integrate the MRI data with PET and obtain attenuation maps. Although optimal accuracy can be expected for specific tissues, such as soft tissue and fat, the characterization of bone and air remains challenging, as they both have near-zero signal intensities. Recently, the precompiled atlas of CT and MRI data has been used to obtain pseudo-CT images, which can be converted to PET attenuation maps. The MRAC is no longer a limitation for PET/MRI application in clinical practice. However, MRAC underestimates SUV, and caution is recommended when comparing SUV values between PET/CT and PET/MRI [1].

## 3. Cervical Cancer

### 3.1. Epidemiology

Uterine cervical cancer is the fourth most common cancer worldwide, with roughly 604,127 cases diagnosed and an annual mortality of 341,831 [4,5]. About 80–90% of the cases described are encountered in developing countries due to the lack of proper screening practices [6]. On the other hand, the incidence has drastically reduced in the United States due to robust screening with pap smear exams and Human Papilloma Virus (HPV) DNA testing and cases hae remained stable during the recent decade (2009–2018). It is estimated that 14,100 new cases and 4280 deaths of invasive cervical cancer will be observed in the United States in 2022 [7]. The survival rate, in general, has been reported as 66%. However, it is lower (39%) in African American women of age ≥65 years [7].

### 3.2. Classification

Most cervical cancers arise from the junctional zone between the cervix’s outer squamous and inner columnar epithelial lining. According to World Health Organization (WHO) classification, cervical cancer can be of various histologic subtypes: (i) squamous cell carcinoma (SCC), (ii) adenocarcinoma, (iii) clear cell adenocarcinoma, (iv) adenosquamous carcinoma, (v) serous carcinoma, (vi) glassy cell carcinoma, (vii) adenoid basal carcinoma, (viii) adenoid cystic carcinoma, (ix) undifferentiated carcinoma, and (x) adenocarcinoma [8]. The SCC constitutes 75% of cervical cancer encounters, while the adenocarcinoma comprises 10–25%, adenosquamous 20%, and the rest of the histologies <5% of cases [6,8]. The dysplastic lesions of SCC can be divided into high-grade squamous intraepithelial lesions (HGSIL) and low-grade squamous intraepithelial lesions (LGSIL).

### 3.3. Imaging

**Figure 1:**
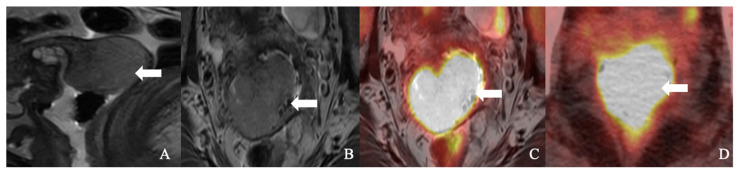
A 51-year-old woman with squamous cell carcinoma of the cervix. (**A**). Sagittal T2-weighted imaging (T2WI), (**B**). axial T2WI, and (**C**). axial fused T2WI positron emission tomography/MRI showing a large (18)F-fluorodeoxyglucose (FDG) avid cervical mass (arrow). (**D**). An axial positron emission tomography/computed tomography image showed FDG avidity cervical tumor (arrow).

Although the previous FIGO classifications did not include the imaging criteria for tumor staging, the 2018 classification has permitted the utility of imaging, which enabled better tumor assessment and staging [5,9,10]. The FIGO staging was based on clinical evaluation due to a limited access to imaging in low-income countries with high cervical cancer prevalence [11]. However, clinical staging is suboptimal for certain tumor characteristics such as size, parametrial invasion, and lymph node involvement. In patients with early-stage cervical cancer (IA and part of IB1), the microinvasion is only detectable using tissue evaluation [12]. The rest of the tumor stages, including local extension, can be assessed using reliable imaging modalities such as CT, MRI, and PET/CT, which have higher sensitivity and comparable specificity to the clinical evaluation [13,14]. The National Comprehensive Cancer Network 2022 Practice Guidelines in Oncology recommended CT or PET/CT for tumor surveillance and follow-up, and MRI for the local assessment of the stage ≥ IB1 [11,15]. Staging is essential to predict survival, and surgical planning is considered standard management for early-stage (≤IIA) cervical cancers [16]. Nguyen et al. compared PET/CT and PET/MRI and found that both modalities could identify all the primary and metastatic lesions and could strongly correlate standardized uptake value (SUV) (*p* = 0.03) [15] (Figure 1).

In general, diffusion weighted imaging (DWI) is considered sensitive to assessing parametrial involvement. It has high false-positive rates if the patients have large tumor sizes or a superimposed infections. Imaging must be highly specific to demonstrate the local tumor invasion since the curative surgery can be performed based on the parametrial invasion [16]. Moreover, identifying stromal, ovarian, or corpus invasion is crucial as they are risk factors for lymphovascular space invasion (LVSI) and para-aortic lymph nodal metastases [17,18].

The successful integration of PET and MRI enabled tumor evaluation and staging in a “one-stop” approach. In a study by Steiner et al., PET/MRI has proven to have a benefit over MRI with an Area Under Curve (AUC) of 0.85 vs. 0.74 for vaginal invasion and 0.89 vs. 0.73 for parametrial invasion [16]. Similar findings were observed in the study by Sarabhai et al., who reported that PET/MRI and MRI are similar in characterizing the T-stage of the tumor (85% vs. 87%) [19]. Wang et al. reported that PET/MRI has a sensitivity, specificity, and negative predictive value (NPV) of 78.5%, 64.9%, and 74.5%, respectively, compared to MRI [20]. PET/MRI characterizes the parametrial invasion with a sensitivity and specificity of 90% and 94% [20]. Kitajima et al. reported a diagnostic accuracy of 83% compared to MRI alone in a study comprising 30 patients [21].

All the studies above were based on morphological observations on PET/MRI. Instead, Wang et al. quantified the gray level values to evaluate the parametrial invasion. They reported that high gray values corresponded to the higher FIGO stages (*p* < 0.05); hence, this quantification technique is practical to implement in clinical practice [20]. Wang et al. described the sensitivity, specificity, and NPV of combined PET/MRI+ gray values as 87%, 84%, and 86%, respectively, compared to MRI or PET/MRI alone, in assessing parametrial invasion (*p* < 0.05) [20].

**Figure 2:**
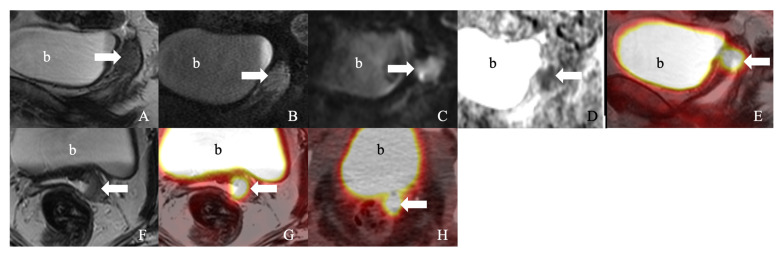
A 55-year-old woman with squamous cell carcinoma of the cervix, status post hysterectomy. (**A**). Sagittal T2-weighted imaging (T2WI), (**B**). post-contrast sagittal T1-weighted imaging (T1WI), (**C**). coronal diffusion-weighted image (DWI), (**D**). coronal apparent diffusion coefficient (ADC), (**E**). coronal fused T2WI, and (**F**). axial T2WI. (**G**). Axial fused T2WI positron emission tomography/MRI showed an enhancing (18)F-fluorodeoxyglucose (FDG) avid plaque-like thickening at the left cervix (arrow) with restricted diffusion. (**H**). An axial positron emission tomography/computed tomography image showed ill-defined FDG avidity (arrow). b: urinary bladder.

The tumor cells drain from the cervix, through the lymphatic vessels, into parametrial lymph nodes, pelvic sidewall nodes, external and internal iliac nodes, and para-aortic nodes [22]. Around 10–30% of patients with cervical cancer demonstrate pelvic lymph node metastases (LNM) during an early stage. This reduces the 5-year survival rate from 94.1% (negative LNM) to 64.1% (positive LNM) [23]. Accurate lymph nodal assessment is essential for developing the individualized treatment algorithm, enhancing the prognosis, and reducing mortality. According to FIGO 2018 classification, micro- or macro-metastases to the lymph nodes are staged as IIIC regardless of tumor size or extent [24]. CT and MRI are less sensitive and specific in detecting metastatic lymph nodes, as they cannot differentiate metastatic from non-metastatic lymph nodes [25,26]. The combined PET/CT was studied, which showed high sensitivity (91% vs. 37.3%) and diagnostic accuracy (98% vs. 95%) compared to MRI (*p* < 0.034), and hence is recommended by the National Comprehensive Cancer Network clinical guidelines [26,27]. However, the PET is limited by identifying small lymph nodal metastases of size < 5 mm [28]. Later, PET/MRI was found to have improved diagnostic confidence over PET/CT with the advantage of a reduced radiation dose [29] (Figure 2). PET/MRI has a sensitivity, specificity, and diagnostic accuracy of 91%, 94%, and 93% in detecting nodal metastases [15]. Compared to PET/CT, PET/MRI identifies nodal metastases with a sensitivity, specificity, and accuracy of 92.3%, 88.2%, and 90%, respectively [21].

Cervical cancer is one of the tumors that demonstrate heterogeneity to hypoxia. Narva et al. studied the association between hypoxia and increased resistance to chemotherapy and radiotherapy in patients with SCC of the cervix [30,31]. In addition, the cancerous cells adapt to the hypoxic microenvironment, leading to genetic instability, DNA damage, and mutagenesis. This results in a rapid tumor invasion to the adjacent and distant organs. ^18^Fluorine-labeled 2-(2-nitro-1-*H*-imidazol-1-y)-*N*-(2,2,3,3,3-pentafluoropropyl)-acetamide (^18^F-EF5) is a hypoxia radiotracer that can be used in PET imaging. Increased uptake of ^18^F-EF5 is strongly associated with poor prognosis compared to (18)F-fluorodeoxyglucose (^18^F-FDG) uptake. Narva et al. reported that an increased ^18^F-EF5 uptake on ^18^F-EF5-PET/MRI correlates with hypoxia intensity, which is proportional to the tumor stage [30].

Radiotherapy is the cornerstone in the management of patients with cervical cancer. Around 25% of cervical cancer cases recur, and 24% among those are observed in already-treated patients, which points to the importance of identifying the radio-resistant tumor areas that may be managed with radiation dose escalation. A new PET tracer ^68^Ga-NODAGA-E[c(RGDyK)]2 ([^68^Ga] (Ga-RGD)) identifies the α_v_β_3_, an integrin that is found on the newly formed vasculature. Pelvic insufficiency fractures (PIF) are a late complication of radiotherapy, and Sapienza et al. studied the incidence of PIF in patients who underwent radiotherapy for various gynecologic cancers [32]. They found that 10–18% of patients are affected by PIF, with the sacrum as the most common fracture site [32]. Azumi et al. noticed PIF in 20% of patients with cervical cancer treated with radiotherapy [33]. They also demonstrated that PET/MRI discovers PIF earlier than PET/CT (*p* <0.05), with the added advantage of reduced radiation exposure [33]. The earliest sign of PIF is medullary edema, which can be observed as T1 hypointense and T2 hyperintense on MRI as early as 18 days after the symptom onset [33].

The maximum standardized uptake value (SUV_max_) derived from [^18^F] FDG-PET and diffusion metrics such as the apparent diffusion coefficient (ADC) from the MRI are studied as the prognostic indicators in patients with cervical cancer [31,34,35,36,37,38,39,40]. Many studies reported that SUVmax and ADC minimum (ADC_min_) values of cervical cancer are inversely related. Olsen et al. also described reduced ADC value in intense SUV_max_ [41]. In addition, the SUVmax was seen to vary based on the histology and degree of differentiation of cervical cancer, and this feature aided in the prognostication [42]. SCC of the cervix is found to have higher SUV_max_ values than the non-squamous tumors (*p* = 0.153), and poorly differentiated ones have higher SUVmax than do the well-differentiated tumors (*p* = 0.0474) [42]. The underlying reason for the difference in SUVmax is secondary to the degree of Glucose Transporter (Glut) expression that aids in FDG uptake; however, it still needs to be validated through further studies [43,44].

The simultaneous acquisition of PET/MRI provides precise spatial correlation and a more appropriate insight into the imaging biomarkers on the voxel level. The inverse correlation between SUV_mean_, SUV_max_, and ADC_min_ was also supported by Brandmaier et al. on hybrid PET/MRI. The correlations between SUV_mean_ and ADC_min_ (*r* = −0.403) and SUV_max_ and ADC_min_ (*r* = −0.532) were significant in primary cervical tumors [45]. The authors demonstrated a stronger correlation between SUV_mean_ and ADC_min_ (*r* = 0.773) and SUV_max_ and ADC_min_ (*r* = −0.747) in the case of recurrent cervical tumors [45]. Grueneisen et al. reported significant SUVmax and ADC_min_ in primary tumors but not the recurrent cervical tumors [46]. Later, Ho et al. described no correlation among SUV_max_, SUV_mean_, ADC_min_, or ADC_mean_. However, they found that the ratio of ADC_min_/ADC_mean_ (relative admin) and the ratio of SUV_max_ and SUV_mean_ (relative SUV_max_) correlated well with the adeno- and adenosquamous carcinoma of the cervix (*r* = −0.685) and with the well- to moderately differentiated tumors (*r* = −0.631) [47]. No significant correlation between relative SUVmax and relative ADC_min_ was found in squamous cell carcinoma and poorly differentiated tumors [47]. Surov et al. studied the SUV and ADC parameters and their relation with the KI 67 proliferation index [48]. They found that SUVmax (*r* = 0.59), SUV_mean_ (*r* = 0.45), SUV_max_/ADC_min_ (*r* = 0.71), SUV_max_/ADC_mean_ (*r* = 0.75), and ADC_min_ (*r* = −0.48) correlated significantly with the KI 67 proliferative index, thereby reflecting the tumor proliferation rate [48]. Additionally, SUV_mean_ (*r* = 0.71) and SUV_max_ (*r* = −0.71) strongly correlate with epithelial and stromal areas and locate the metabolically active areas [48]. In addition to SUV and ADC, the other parameters include metabolic tumor volume (MTV) and total lesion glycolysis (TLG). It has been studied that these parameters conventionally correlate with the SCC antigen levels, FIGO staging, tumor size, and depth of stromal invasion [49,50]. Table 3 and Table 4 summarize the essential characteristics of PET/MRI studies in cervical and pelvic malignancies.

## 4. Endometrial Cancer

### 4.1. Epidemiology

Cancer of the uterine corpus comprises endometrial and myometrial cancers [65,66]. The majority of the tumors are adenocarcinomas from the endometrium and sarcomas from the myometrium. Around 65,950 new cases and 12,550 deaths due to cancer of the uterine corpus are estimated in 2022 by the American Cancer Society [2]. These cancers are often referred to as endometrial cancer, as >90% of the cases demonstrate malignancy in the endometrial lining [65]. It is observed among 7% of cancer patients [66].

### 4.2. Classification

Of all the endometrial carcinoma histologies, endometroid adenocarcinoma accounts for 75% of the endometrial cancers, while mixed, uterine papillary serous, clear cell, carcinosarcoma, mucinous, squamous cell, and undifferentiated comprise 10%, <10%, 4%, 3%, 1%, <1%, and <1%, respectively [66]. Endometrial cancer can be classified as type I (80–90% of cases) and type II (10–20% of cases) carcinomas [67]. Type I is low-grade (grades 1 and 2) endometroid adenocarcinoma arising from complex atypical hyperplasia and is secondary to unopposed estrogen exposure. Type II arises from atrophic endometrium and comprises high-grade endometroid adenocarcinoma (grade 3) and non-endometroid histologies. Type II tumors are clinically aggressive and associated with poor prognosis. FIGO staging of the cancer also plays a vital role in estimating the prognosis. The “new risk groups to guide adjuvant therapy use” were described during the recent conference European Society for Medical Oncology-European Society of Gynecological Oncology-European Society for Radiotherapy and Oncology (ESMO-ESGO-ESTRO) [68]. The consensus stratified stages IA of grades 1–2 and IB endometroid adenocarcinomas of grades 1–2 without LVSI as low and intermediate risk groups. At the same time, Stage ≥ II, grade 3 endometroid adenocarcinomas, with LVSI, or non-endometroid tumors, are categorized under high-intermediate or higher risk groups [69]. The 5-year overall survival (OS) for Stages I, II, III, and IV ranges between 85 and 90%, 75 and 85%, 50 and 56%, and 20 and 25%, respectively [67].

### 4.3. Imaging

The role of imaging-derived biomarkers in endometrial cancer was discussed at the ESMO-ESGO-ESTRO consensus conference, highlighting the relevance of imaging in surgery planning [68]. Integrated PET/MRI has been studied to identify cancer prognosis through the individual biomarkers (i) SUV and (ii) ADC. The increased SUV on the PET scan indicates the tumor’s strong glucose metabolism and represents the tumor’s aggressiveness. The ADC value on DWI is obtained based on the diffusion of the water molecules. Its value is reduced in the tumor tissue secondary to high cellularity. Shih et al. described the inverse correlation between SUV_max_ and ADC_min_ and reported that these components are associated with poor pathological prognostic factors [70]. A more recent study by Tsuyoshi et al. reported that SUV cannot differentiate between low-and high-risk endometrial cancers and cannot be relied on to establish the prognosis. The same study demonstrated that lower ADC values (*p* < 0.05) and a higher SUV-to-ADC ratio (*p* < 0.005) are associated with an increased risk of cancers. An SUV-to-ADC ratio of 16.9 × 109 predicts tumor aggressiveness with a sensitivity, specificity, and accuracy of 73%, 81%, and 77%, respectively [69].

The depths of myometrial invasion and lymphovascular space invasion (LVSI) aid in the extent of the surgical intervention [71]. With its enhanced soft tissue evaluation, MRI provides information on the importance of myometrial involvement. The PET imaging assesses the LVSI and correlates well with endometrial tumor prognosis. Combined PET/MRI has been shown to possess an excellent positive predictive value to evaluate both myometrium and LVSI [67]. Ironi et al. reported that PET/MRI has a sensitivity, specificity, accuracy, PPV, and NPV of 85%, 92%, 91%, 75%, and 96%, respectively, in detecting LVSI [67]. At the same time, the parameters were 72%, 84%, 77%, 88%, and 64%, respectively, in the evaluation of the myometrial invasion [67]. The conventional entire field of view (fFOV) DWI is usually performed by single-shot echo-planar imaging (ssEPI). To focus on the region of interest (ROI), a reduced FOV (rFOV) ssEPI technique termed as an optimized and constrained undistorted single shot (FOCUS) is described using the spatial radiofrequency pulse signals that are limited to the ROI [72]. A study by Ota et al. reported that the high-resolution rFOV DWI, compared to fFOV DWI, has fewer distorted images, with enhanced image quality and diagnostic performance to evaluate the deep myometrial invasion of endometrial cancer [72]. Table 5 summarizes the essential characteristics of PET-MRI studies in endometrial malignancies.

## 5. Ovarian Cancer

### 5.1. Epidemiology

Ovarian cancer is the fifth leading cause of cancer-related deaths in women and accounts for 2.1% of all cancer-related deaths [78,79]. It is usually diagnosed in women aged ≥63 years and is more common in white than African American women [78]. Approximately 1 in every 78 women is at risk of ovarian cancer [78]. According to 2022 cancer statistics, around 19,880 new cases and 12,810 ovarian cancer deaths are estimated to occur in the United States [7]. This incidence has reduced by 1–2% per year between 1990 and 2010. However, it has increased by 3% annually from 2010 to 2018 [7]. Only 19% of ovarian cancers are discovered at an early localized stage and are associated with a 5-year survival rate of 93% [7]. The rest of the patient population has often delayed presentation due to the silent nature of the disease, which is responsible for a reduced 5-year survival rate in the same population (49%) [7]. Age is another critical factor that affects the survival rate. The survival rate is 61% in women aged <65 compared to 33% in women aged ≥65 [7].

### 5.2. Classification

Ovarian tumors can be classified into epithelial, mesenchymal, sex-cord stromal, and germ-cell tumors. Of all the types, malignant epithelial tumors constitute 60% of all ovarian and 90–98% of malignant ovarian neoplasms [80,81]. High-grade serous ovarian carcinoma (HGSC) (70%), low-grade serous ovarian carcinoma (LGSC) (<5%), clear cell carcinoma (10%), endometroid carcinoma (10%), and mucinous carcinoma (1.5–3%) are the five subtypes of malignant epithelial neoplasms [80]. It is pivotal to differentiate the HGSC and LGSC on imaging, as they possess distinct molecular pathogenesis and treatment responses. HGSC has diffuse peritoneal involvement associated with peritoneal deposits and large-volume ascites on imaging [82]. They have been studied to arise from the tubal intraepithelial cells, also termed “serous tubal intraepithelial carcinoma.” The LGSC has an indolent clinical course with progression from the benign tumor (serous cystadenoma), atypical proliferation (serous borderline), carcinoma in situ, and LGSC. Unlike HGSC that are associated with TP53 (73–96%) and BRCA ½ (11–12%) mutations, LGSC is frequently associated with KRAS (0–55%) and BRAF (0–38%) mutations [82,83]. Brenner tumors and seromucinous carcinoma were also categorized under ovarian neoplasms in the most recent FIGO classification in 2014 [83].

### 5.3. Imaging

Imaging plays a crucial role in the management of ovarian cancer. Ultrasound (US) provides basic information on the presence of an ovarian mass and demonstrates the benign or malignant features. It is a sensitive imaging modality (97%). However, it lacks specificity (71%), which may lead to unnecessary surgical intervention [84,85]. CT is the next imaging modality that provides decisive interpretation regarding tumor staging, treatment response, and recurrence. It also aids in identifying lymph node and organ metastasis with superior sensitivity than US.

As per the European Society of Urogenital Radiology (ESUR), MRI and contrast-enhanced MRI are the alternative modalities in patients with indeterminate adnexal lesions in US and CT [86,87]. It has a sensitivity, specificity, and accuracy of 83%, 84%, and 83%, compared to CT or US, in evaluating indeterminate ovarian lesions [88]. These values increase to 95%, 98%, and 97%, respectively, if the ADNEX MR scoring system is used to evaluate the adnexal lesions [89]. The ADNEX MR scoring system is the standard of imaging evaluation, similar to the Breast Imaging Report Data System (BI-RADS). The lymph node or distant organ metastases can be easily identified on 18F-FDG-PET/CT compared to US, CT, or MRI. However, the false-positive reports are higher due to the increased uptake of the FDG by the normal ovaries in the late follicular to early luteal phase [90,91]. In addition, FDG-PET/CT has low diagnostic value in differentiating early low-grade carcinoma and borderline tumor due to the low FDG uptake, which results in a false-negative value [90].

**Figure 3:**
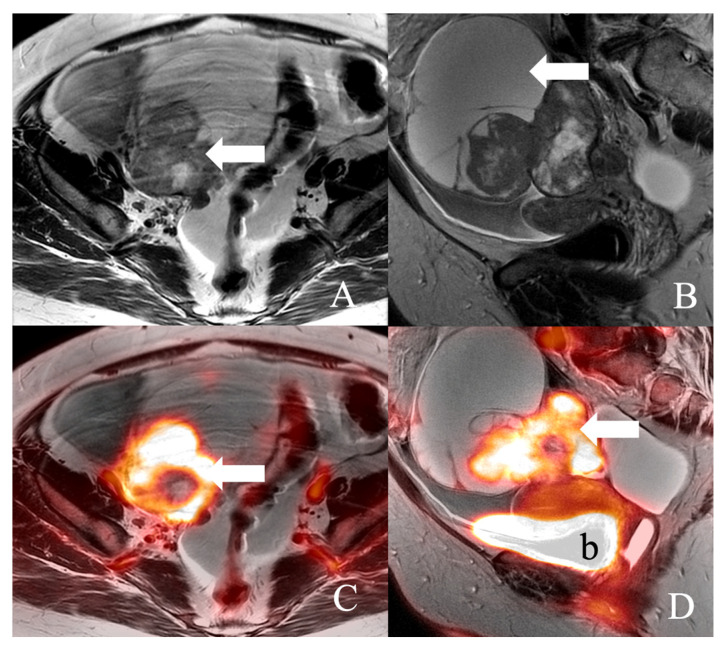
49-year-old female with high-grade Mullerian carcinoma of the right ovary. (**A**) Axial T2WI, (**B**) Sagittal T2WI MR images, and (**C**) Axial fused T2WI. (**D**) Sagittal fused T2WI positron emission tomography/MRI images demonstrate a large complex right adnexal mass (arrow) with an enhancing soft tissue component within the mass. b, urinary bladder.

Combined, PET/MRI has proven to be helpful in the characterization of ovarian tumors with a sensitivity, specificity, PPV, and NPV of 94%, 100%, 100%, and 83%, respectively, compared to individual PET/CT and MRI [92] (Figure 3). The fusion PET/MRI is superior to PET/CT due to the excellent soft tissue resolution, which aids in identifying even minute hypermetabolic tumor masses. Similar results were reported by Fiaschetti et al., who described the sensitivity and specificity of MRI, PET/CT, and PET/MRI as 84% and 60%, 74% and 80%, and 94% and 100%, respectively [92]. However, a study by Tsuyoshi et al. reported that PET/MRI has similar efficacy to contrast-enhanced CT or contrast-enhanced MRI in determining the T-stage of the ovarian tumor [93]. The same study described PET/MRI as superior to contrast-enhanced CT in the M-staging and which can be used as an alternative [93].

Cytoreductive surgery is the standard of care for patients with ovarian cancer [86]. In patients where complete debulking is not feasible, neoadjuvant chemotherapy (NAC) and interval debulking surgery are the optimal therapeutic choices. The response of the tumor to NAC is assessed by monitoring the CA-125 levels before and after the NAC [94]. PET/CT has a proven benefit in predicting the treatment response by measuring before and after the NAC [95]. However, the role of PET/MRI has been established to assess the tumor response in cervical cancer, and its capability is questionable in the case of ovarian tumors [93]. Table 6 and Table 7 summarize essential characteristics of PET-MRI studies in ovarian and gynecological malignancies.

Around 80% of patients respond well to debulking surgery and NAC [101]. Unfortunately, 75% of these patients relapse within two years [101]. The recurrence rate also depends on the tumor’s initial stage and is 10%, 30%, 70–90%, and 90–95% in patients with Stages I, II, III, and IV ovarian cancer [102]. Early recognition of the recurrence is essential in planning the optimal treatment, and PET/MRI has proven to have an excellent diagnostic performance in recurrence detection. In a meta-analysis by Zheng et al., PET/MRI had a sensitivity and specificity of 96% and 95% in restaging patients suspected of having a pelvic malignancy recurrence, including ovarian tumors [103]. Specific to ovarian malignancies, PET/MRI has comparable sensitivity, specificity, and accuracy to contrast-enhanced CT in determining the recurrence (100%, 100%, and 100%, respectively, vs. 89%, 100%, and 91%, respectively) [93].

## 6. Challenges

Physiologic variations in FDG uptake may mimic disease, resulting in misdiagnosis or inaccurate staging. Conditions such as seromas, abscesses, ovarian hyperstimulation, and fibroids can cause false results. The assessment of lung metastases can be challenging in PET/MRI. Chandarana et al. reported that MRI with simultaneously acquired PET data has high sensitivity in detecting FDG-avid lung nodules (70.3% vs 61.6%, *p* = 0.002) and nodules with a diameter of at least 0.5 cm (81.8%) [104]. Acquiring PET/MRI requires technologists to have dual training in PET and MRI. Having two technologists present, each with one of these two proficiencies, may solve this problem, but will be costlier. Another issue relates to the lack of reimbursement for PET/MRI services. There are also no specific Current Procedural Terminology (CPT^®^) codes for PET/MRI for reimbursement. As such, this requires submitting individual codes for whole-body PET and MRI.

## 7. Clinical Trials and Metanalysis

A list of the currently ongoing clinical trials regarding the diagnostic utility of PET/MRI in gynecological malignancies can be found in Table 8. These trials are recruiting participants as of the time of writing this manuscript and hopefully will provide better larger-scale data regarding the use of PET/MRI in such patients. Few metanalyses studies have also been conducted with encouraging results, as mentioned in Table 9.

## 8. Future Directions

Recently, it has been reported that ADC value does not accurately reflect the diffusion of water molecules, as it is based on just a Gaussian distribution model [108]. Hence, diffusion kurtosis imaging (DKI) has been developed, which measures the non-Gaussian distribution, thereby reflecting the tissue microstructure [108]. The application of DKI has been well-studied in gliomas, prostate cancers, and hepatic fibrosis [109]. A pilot study by Wang et al. demonstrated that DKI has the ability to differentiate the stage and grade of uterine cervical cancer [110]. In patients with endometrial cancer, Chen et al. and Yue et al. have proven that DKI is more feasible than DWI in distinguishing high from low grade endometrial cancers [111,112]. Among patients with ovarian tumors, although DKI correlates with Ki-67 expression, it did not demonstrate any added advantage over DWI in a study by Li et al. [113]. As the DKI is still a research tool and only few studies support its application, it is at a stage where it can be analyzed in a broader clinical setting.

Machine learning (ML) is another revolutionary concept in the oncological field. It is a subset of artificial intelligence and aids in the diagnosis, treatment, prognosis, and clinical decision making of various cancer types. Multiple ML algorithms have been reviewed in gynecologic oncology. For instance, Lawresnon et al. studied ML in elucidating the cellular origin of HGSC [114]. Authors identified that HGSC has a dual cellular origin from ovarian surface epithelial and fallopian secretory epithelial cells [114]. ML predicts the prognosis and stratifies the high-risk patients diagnosed with cervical or endometrial cancers [115,116,117]. In the near future, sufficient authorization of the blooming ML methods/algorithms will lay the support for precision medicine in gynecologic cancers.

## 9. Conclusions

MRI and PET are both essential in the diagnosis and surveillance of gynecological malignancies and providing complementary knowledge about local tumor staging, metastases detection, and recurrence evaluation. Integrating them into a single “one-stop” examination via PET/MRI has clear advantages in terms of patient convenience, radiation dose reduction, and the imaging of pelvic malignancies.

## Figures and Tables

**Table 1:** 3T MRI pelvis protocol sequences.

Sr. No.	Series Description	Field of Vision	Slice Thickness	Spacing	Frequency Encoding	Frequency × Phase
1	Coronal T2WI	420	5	0	Superior/Inferior	288 × 192
2	Sagittal T2WI	240	5	0	Anterior/Posterior	320 × 224
3	FOV Sagittal b = 50, 600	240	5	0	Superior/Inferior	96 × 80
4	Axial T2WI	240	5	0	Left/Right	320 × 224
5	Axial T1WI	240	5	0	Left/Right	320 × 224
6	Axial Diffusion b = 50, 400, 800 Sagittal Diffusion pelvis 0, 600	380	5	0	Left/Right	96 × 160
7	Axial 3D pre-contrast T1WI	240	5	−2.5	Left/Right	320 × 224
8	Dynamic	240	5	−2.5	Superior/Inferior	256 × 224
9	Axial 3D post-contrast T1WI immediate delay	240	5	−2.5	Left/Right	320 × 224

WI: weighted images; FOV: field of vision; Sr No: serial number; 3D: 3-dimensional.

**Table 2:** PET/MRI pelvis protocol sequences.

PET/MRI Sequences
Focused Pelvis (performed first).
Fused axial 3D T1WI LAVA.
Fused sagittal T2WI FSE.
Fused axial T2WI FSE.
Fused post-contrast axial T1WI.
Whole body (performed post-contrast after focused pelvis)
Auto-bind axial 3D T1WI LAVA water images.
Fused axial 3D T1WI LAVA.
Fused sagittal 3D T1WI LAVA (reformat).
Fused coronal 3D T1WI LAVA (reformat).
MIP PET MAC

FSE: fast spin echo; MAC: measured attenuation correction; MIP: maximum intensity projection; 3D: 3-dimensional, LAVA: liver acceleration volume acquisition.

**Table 3:** Characteristics of PET/MRI studies in cervical cancer.

Serial Number	Study	Year of Publication	Type of Study	Total Patient Number	Objective of Study	PET MRI Machine Details	Result	Limitations
1	Floberg et al. [51]	2018	Retrospective	17	To describe the relation between ADC and SUV values on MRI and PET imaging, respectively.	nMR-integrated PET/MRI	SUV_mean_ and ADC_mean_ (*p* = 0.007) and SUV_mean_ and ADC_T/M_ (*p* = 0.008) are inversely correlated. Such inverse correlation was not statistically significant when the tumors were divided into Adenocarcinomas and SCC.	Retrospective study with small sample size; Heterogeneous patient cohort including patients treated with surgery or chemoradiation and cancers of varied sizes, grades, histology, and stages.
2	Nguyen et al. [15]	2020	Prospective	6	To compare the diagnostic performance of FDG PET/MRI vs. PET/CT.	Discovery 710 PET/CT and Biograph mMR 3T scanner	There is a strong correlation between the tumor SUVs on PET/CT and PET/MRI (*p* < 0.001). PET/MRI has superior diagnostic interpretation and identified 4 of the 6 tumors not identified on PET/CT.	Small sample size; Lack of histological confirmation and correlation; Confounding bias as a result of the time gap between the two imaging methods
3	Surov et al. [48]	2017	Prospective	21	To study the relation between ADC and SUV values, and their importance in estimating tumor proliferation (KI 67).	Biograph mMR PET/MRI	SUV_max_ (*p* = 0.005), SUV_mean_ (*p* = 0.04), ADC_min_ (*p* = 0.03), SUV_max_/ADC_min_ (*p* = 0.001), and SUVmax/ADC_mean_ (*p* = 0.001) are significantly correlated with KI-67	Small sample size
4	Anner et al. [26]	2016	Retrospective	27	To study the quality of MRI, PET/CT, and PET/MRI in the lymph nodal staging of cervical carcinoma. Authors compared the diagnostic efficacy of imaging compared to histological analyses.	64-row multidetector PET/CT, Magnetom trio 3-T MRI; PET/MRI images were reconstructed virtually from individual MRI and PET/CT images	PET/MRI has similar sensitivity (64%) and moderate specificity (77% vs. 69%), PPV (75% vs. 69%), and NPV (67% vs. 64%) compared to PET/CT images. Hence, the study concluded that PET/MRI is not superior to PET/CT in the lymph nodal staging of cervical cancer patients.	Small population; Retrospective study design; Discrepancy between the imaging and histological analyses; Virtually reconstructed PET/MRI images rather than originally obtained scanner images.
5	Wang et al. [20]	2019	Retrospective	79	To study the diagnostic efficacy of integrated PET/MRI in identifying the parametrial involvement and the importance of gray value while interpreting PET/MRI.	Signa PET/MRI (Integrated scanner)	The accuracy, sensitivity, and NPV of PET/MRI are higher than conventional MRI; however, it was not significant (*p* = 1.0). The accuracy, sensitivity, and NPV of combined PET/MRI+ gray values are significantly superior to conventional MRI (*p* < 0.05).	Retrospective analysis resulting in selection bias; Small sample size; No evaluation between multiple observers.
6	Narva et al. [30]	2021	Prospective	9	To evaluate the correlation between PET/MRI imaging (^18^F-EF5) and endogenous hypoxia (such as HIF1, CAIX, and GLUT1) tracers.	Ingenuity TF PET/MRI	18F-EF5 max T/M ratio (*p* = 0.036) and HSV (*p* = 0.040) correlated with advanced-stage tumors and HSV correlated with tumor size (*p* = 0.02).	Small sample size; the chemistry of EF5 is complex, which may limit its broad application.
7	Brandmaier et al. [45]	2015	Prospective	31	To study the correlation between ADC and SUV values on simultaneous PET/MRI and their importance in primary and recurrent cervical cancer.	Magnetom Biograph mMR PET/MRI scanner	There was a significant inverse correlation between ADCmin and SUVmax (*p* = 0.05) and SUVmean and ADCmin (*p* = 0.03) in patients with primary tumors, primary metastases, and recurrent tumors (*p* = 0.002); No significant correlation among patients with recurrent metastases (*p* > 0.05).	Histopathological correlation was not performed; Included are the visible lesions on both imaging modalities; Average uptake time for FDG on PET/MRI is approximately 30 min, which could affect the SUV measurements.
8	Umutlu et al. [52]	2020	Prospective	30	To evaluate if PET/MRI can identify N- and M-staging of primary cervical cancers and, based on the results, if it can be a platform for radiomics analysis and artificial intelligence algorithms.	Biograph mMR PET/MRI scanner	PET/MRI is superior in determining the M-stage than the N-stage, with a sensitivity and specificity of 91% and 92%, respectively. AUC was 0.97 for the M-staging and 0.82 for the N-staging.	Small patient cohort; Heterogeneous histopathology and tumor sizes.
9	Meyer et al. [53]	2018	Prospective	18	To study the correlation between the parameters of cervical cancer’s histopathology and PET/MRI imaging.	Biograph mMR PET/MRI scanner	Authors identified no significant correlation between SUVmax, SUVmean, and ADC histogram parameters; Total lesion glycolysis was correlated inversely with p25, p75, p90, ADCmedian, and ADCmode. MTV also significantly corelated with ADCmean, p10, p25, p75, p90, ADCmedian, and ADCmode.	Retrospective study; Small sample size; Only squamous cell carcinomas were evaluated.
10	Sarabhai et al. [19]	2017	Prospective	53	To compare the efficacy of PET/MRI and MRI alone for evaluating primary and metastatic cervical tumors.	Biograph mMR whole-body PET/MRI scanner	T-staging: PET/MRI vs. MRI alone classified 85% vs. 87% of tumors (*p* > 0.1); N-staging: Sensitivity, specificity, and accuracy of PET/MRI were 83%, 90%, and 87%, respectively, and that of MRI alone were 71%, 83%, and 77%, respectively (*p* > 0.05); M-staging: Sensitivity, specificity, and accuracy of PET/MRI were 87%, 92%, and 91%, respectively, while that of MRI alone were 67%, 90%, and 83%, respectively (*p* > 0.05).	Small patient cohort and statistical power; Authors used restricted reference standards for all suspicious lesions.
11	Steiner et al. [16]	2021	Retrospective	33	To compare the efficiency of PET/MRI and MRI alone; Role of ADC and SUV values in primary cervical cancer.	Hybrid 3T Ingenuity TF PET/MRI scanner on a phased-array SENSE XL	PET/MRI has higher AUC compared to MRI alone in detecting deep stromal invasion (0.96 vs. 0.74), parametrial invasion (0.89 vs. 0.73), and vaginal invasion (0.85 vs. 0.74); PET/MRI is more sensitive than MRI alone in ruling out residual tumors after radical cone biopsy or hysterectomy (89% vs. 44%); PET/MRI has equal AUC to MRI alone in pelvic nodal staging (0.73 vs. 0.73) but not distant metastases (0.80 vs. 0.67).	Retrospective study; Small cohort; ADC values were obtained from ROI-based mean, rather than whole tumor volume.
12	Vojtisek et al. [54]	2021	Retrospective	66	To identify the role of PET/MRI in predicting tumor treatment response to chemoradiotherapy.	Biograph mMR PET/MRI scanner	The PET/MRI parameters, including mid-MTV, mid-TLG, mid-TLG-S, mid-MTV-s, mid-tumor size, and change in % SUVmax, were significantly different between the responders and non-responders. Of all the parameters, mid-MTV-s showed moderate discrimination ability to identify non-responders.	Small cohort; Shorter follow-up interval.
13	Ahangari et al. [55]	2021	Retrospective	18	To evaluate the workflow with PET/MRI in cervical cancer patients undergoing radiotherapy.	Biograph mMR PET/MRI scanner	PET images reconstructed with sCT and CT had no significant difference in quantification for all patients.	Residual error due to alignment issues between CT and MRI; As the weight is the limiting factor, one of the patients in the current study did not fit the PET/MRI coil holder.
14	Kim et al. [56]	2009	Retrospective	79	To study the efficacy of fusion PET/MRI in detecting of metastatic lymph nodes in cervical cancer.	Signa 1.5T MRI; Biograph LSO or Discovery LS PET/CT scanner; Images are fused using advantage windows workstation.	PET/MRI has higher diagnostic performance than PET/CT in identifying the metastatic lymph nodes (*p* = 0.0259); In addition, it has sensitivity and specificity of 54% and 93%, respectively, and that of PET/CT are 44% and 94%, respectively.	Verification bias as the surgeons are guided by pre-operative MRI and PET/CT; Node-by-node comparison was not performed; instead, the notable lymph node identified grossly or on imaging was considered.
15	Ahangari et al. [57]	2022	Prospective	10	To study the role of simultaneous PET/MRI in the characterization of tumor heterogeneity before chemoradiotherapy.	Biograph Vision 600 PET/CT scanner; Biograph mMR whole-body PET/MRI scanner	There was a strong correlation between the SUV and ADC values in patients with cervical cancer (*r* = −0.7).	Small patient population.
16	Azumi et al. [33]	2021	Retrospective	149	To study the risk factors associated with pelvic insufficiency fractures in cervical cancer and the role of PET/MRI in PIF diagnosis.		The pelvic insufficiency fractures were detected earlier on PET/MRI compared to PET/CT (*p* < 0.05).	Retrospective study; Measured SUV values on PET/CT and PET/MRI may differ due to the difference in detectors and reconstruction methods.
17	Gong et al. [58]	2021	Retrospective	114	To study the role of PET/MRI as diagnostic imaging in cervical cancer.	Biograph Truepoint 64-row multidetector PET/CT and Magnetom Biograph mMR PET/MRI	PET/MRI is more sensitive (90–100% vs. 62–67%) and specific (96% vs. 93%) than PET/CT in detecting primary tumors and bladder invasion. The SUVmax and SUVmean values obtained on PET/MRI were higher than PET/CT in patients with primary tumors, bladder involvement, and para-aortic lymph nodal invasion (*p* < 0.001). The difference is insignificant in patients with vaginal (*p* = 0.3) or pelvic lymph node involvement (*p* = 0.4).	Different FDG uptake periods between the PET/CT and PET/MRI were considered; the diagnostic value of lymph nodal size was not analyzed

PET: Positron Emission Tomography; MRI: Magnetic Resonance Imaging; ADC: Apparent Diffusion Coefficient; SUV: Standardized Uptake Volume; ADC_T/M_: SCC: Squamous cell carcinoma; FDG: (18)F-fluorodeoxyglucose; CT: Computed tomography; KI-67: Proliferation Index; PPV: Positive Predictive Value; NPV: Negative Predictive Value; 18F-EF5: ^18^Fluorine-labeled 2-(2-nitro-1-*H*-imidazol-1-y)-*N*-(2,2,3,3,3-pentafluoropropyl)-acetamide; HIF1: Hypoxia-Inducible Factor 1; CAIX: Carbonic Anhydrase; GLUT1: Glucose Transporter; 18F-EF5 T/M ratio: 18F-EF5 Tumor to Muscle uptake ratio; HSV: Hypoxic subvolume; MTV: Metabolic Tumor Volume; AUC: Area Under the Curve; TLG: Total Lesion Glycolysis; mid-TLG-S and mid-MTV-s: Midtreatment parameters at week 5 of chemoradiotherapy; sCT: MRI-derived Synthetic CT; PIF: Pelvic Insufficiency Fracture.

**Table 4:** Characteristics of PET/MRI studies in pelvic malignancies.

Serial Number	Study	Year of Publication	Type of Study	Total Patients in Study	Objective	PET MR Machine Details	Result	Limitations
1	Xin et al. [59]	2016	Prospective	45	To evaluate the diagnostic performance of PET/MRI in abdominal and pelvic tumors compared to PET/CT.	Discovery 690 PET/CT; Ingenuity TF PET/MRI scanner	There was no significant difference in tumor identification on PET/CT and PET/MRI (*p* = 0.18); However, PET/MRI images had better quality than PET/CT; There was an excellent correlation of SUV value to the focal lesions (*R* = 0.948).	PET/MRI was obtained 105 min after PET/CT, which might have led to physical decay and tracer biokinetics; The position of arms varied between the PET/CT and PET/MRI, which could be the reason for the difference in image quality.
2	Queiroz et al. [60]	2015	Prospective	26	To study the role of PET/CT and PET/MRI in staging and re-staging of advanced gynecological cancers.	Discovery PET/CT 690; the fusion was performed on the Advantage workstation	PET/MRI is superior to PET/CT for primary tumor identification (*p* < 0.001). No difference was found in the evaluation of lymph nodes and abdominal metastases.	Small patient population; PET/MRI was not obtained from whole-body imaging.
3	Spick et al. [61]	2016	Retrospective	69	To study whether PET/MRI has improved diagnostic performance in cancer assessment.		PET/MRI has similar diagnostic accuracy as PET/CT in the detection of primary and recurrent pelvic cancers; However, the diagnostic confidence of PET/MRI is higher than PET/CT in benign (*p* < 0.05) and malignant (*p* < 0.01) lesions. In addition, lesion conspicuity was better on PET/MRI compared to PET/CT.	
4	Grueneisen et al. [62]	2014	Prospective	48	To study the role of DWI in PET/MRI imaging for primary and recurrent tumor evaluation.	Biograph mMR 3-T PET/MRI scanner	There was no significant effect of DWI on the diagnostic performance of PET/MRI (*p* > 0.05); In fact, higher diagnostic confidence was noted with PET than with the DWI (*p* < 0.05).	Included 48 patients; however, further studies are required to validate the results. Image and histopathological correlation were performed using restricted reference standards.
5	Schwartz et al. [63]	2018	Prospective	18	To compare the diagnostic ability of PET/MRI to PET/CT for patients with gynecologic malignancy.	Biograph mCT PET/CT scanner; Biograph mMR 3-T PET/MRI scanner	PET/CT and PET/MRI have similar diagnostic potential in visualizing the regional lymph nodes and abdominal metastases, whereas PET/MRI is more sensitive than PET/CT in demonstrating the soft-tissue involvement.	Small cohort; Heterogeneous sample; PET/MRI was limited to only abdominopelvic cavity.
6	Nakajo et al. [64]	2010	Retrospective	31	To compare the diagnostic accuracy of FDG PET/CT vs. PET/MRI in gynecological malignancies.	Fusion of PET/CT and MRI images was conducted using Osirix imaging software	PET/T2W MRI images localized the lesion better than the PET/T1W or PET/CT images during the first (*p* < 0.01), second (*p* < 0.01), and third (*p* < 0.01) evaluation.	Misregistration secondary to motion artifact between PET and MRI; Pelvis MR images, instead of whole-body MR images, were used for fusion images; Only two readers scored the images.

PET: Positron Emission Tomography; MRI: Magnetic Resonance Imaging; ADC: Apparent Diffusion Coefficient; SUV: Standardized Uptake Volume; CT: Computed tomography; FDG: (18)F-fluorodeoxyglucose; DWI: Diffusion Weighted Imaging.

**Table 5:** Characteristics of PET/MRI studies in endometrial cancer.

Serial Number	Study	Year of Publication	Type of Study	Total Patients in Study	Objective	PET MR Machine Details	Result	Limitations
1	Ironi et al. [67]	2021	Retrospective	35	To study the pre-operative diagnostic role of PET/MRI in assessing myometrial and lymph nodal involvement of endometrial cancer.	Signa Hybrid PET/MRI scanner	Lymph node involvement: PET/MRI demonstrated a sensitivity, specificity, accuracy, NPV, and PPV of 85%, 92%, 91%, 96%, and 75%, respectively; Myometrial invasion: PET/MRI demonstrated a sensitivity, specificity, accuracy, NPV, and PPV of 72%, 84%, 77%, 64%, and 88%. The MRI- and PET-derived tumor volume, volume index, tumor–volume ratio, and total-lesion glycolysis were significant predictors of lymph nodal space invasion (*p* = 0.002, 0.006, 0.002, 0.013, respectively).	Small study population.
2	Jonsdottir et al. [73]	2021	Prospective	34	To investigate the efficacy of PET/MRI, in comparison to DWI–MRI, for evaluating peritoneal carcinomatosis in gynecological tumors.	Signa 3T PET/MRI scanner	The PCI of PET/MRI (*p*-0.6) is closer to surgical PCI than DWI–MRI (*p* = 0.007). In addition, PET/MRI is a useful tool that aids in deciding the operability of cancer.	Small cohort of patients; Included the patients who received neoadjuvant chemotherapy and those who were primarily operated on.
3	Tsuyoshi et al. [74]	2020	Prospective	36	To study the role of PET/MRI in pre-operative staging of endometrial cancer.	Signa PET/MRI scanner	PET/MRI is equivalent to contrast-enhanced in assessing nodal and distant metastatic staging (*p* > 0.05); hence, it can be considered an alternative imaging modality during the pre-operative staging of endometrial cancer.	Retrospective study; A few MRI images were not performed at the author’s institution, however, were re-read by the radiologists at the author’s institution; Small population study; Histological correlation was not performed in 36% of patients, as they did not undergo lymphadenectomy.
4	Bian et al. [75]	2019	Retrospective	81	To evaluate the diagnostic performance of PET/MRI and PET/CT for regional lymph node metastases and myometrial involvement in endometrial cancer.	Biograph 64 PET/CT; Biograph mMR PET/MRI scanner	Regional lymph node metastases: PET/MRI has superior sensitivity (*p* = 0.015) and specificity (*p* < 0.001) than PET/CT; Myometrial involvement: Accuracy of PET/MRI is higher than PET/CT (82% vs. 46%).	Patients did not undergo simultaneous PET/CT and PET/MRI; Retrospective study; Small study population.
5	Tsuyoshi et al. [69]	2019	Retrospective	31	To evaluate the role of PET/MRI in determining the phenotypes of endometrial cancer.	Signa 3-T PET/MRI scanne	Lower ADC values (*p* < 0.05) and higher SUV-to-ADC ratio (*p* < 0.005) are associated with high-risk cancers more than low-risk cancers. In turn, the SUV-to-ADC ratio demonstrated higher diagnostic accuracy and higher AUC values (*p* < 0.05); The SUV-to-ADC ratio cut-off value of 16.9 × 10^9^ has a sensitivity, specificity, and accuracy of 73%, 81%, and 77%, respectively, in predicting high-risk cancer groups.	Small sample size; Mean SUV and ADC values at the center of the lesion were considered.
6	Bezzi et al. [76]	2021	Systematic review and meta-analysis		To study the role of PET/MRI in staging and re-staging of endometrial cancer.		PET/MRI has the highest diagnostic accuracy in detecting the soft-tissue involvement and metastases. SUV to ADC ratio seems to be a more reliable index to describe the aggressiveness of endometrial cancer; PET/MRI detects the post-therapeutic changes of the lesion and even small recurrent lymph node lesions.	Small cohort study; Heterogeneous patient population.
7	Stecco et al. [77]	2016	Retrospective	27	To evaluate the clinical utility of retrospective fusion PET/MRI–DWI obtained through individual PET/CT and MRI–DWI.	Biograph 16 HI-REZ PET/CT; Achieva Intera 1.5T MRI scanner	Although on a per-patient basis PET/MRI has similar sensitivity, specificity, and accuracy to PET/CT, PET/MRI is superior in terms of per-node basis. The sensitivity (89% vs. 70%), specificity (92% vs. 91%), accuracy (91% vs. 87%), PPV (69% vs. 60%), and NPV (98% vs. 94%) are better for PET/MRI than PET/CT. There was no significant difference between PET/CT and PET/MRI–DWI in the detection of metastatic lymph nodes.	Small sample size; Pelvic MR images are considered in the study instead of whole-body MRI; Scores were subjective, and only two interpreters were involved in the study.
8	Shih et al. [70]	2015	Prospective	36	To study the correlation between SUV_max_ and ADC_min_, and their roles in determining the prognosis.	Biograph mMR PET/MRI scanner	SUVmax and ADCmin are inversely correlated with each other (*p* = 0.001); Higher SUVmax and lower ADCmin are associated with advanced tumor stages (*p* < 0.05); Study also found that a higher ratio of SUVmax to ADCmin is indicative of advanced tumor (*p* < 0.05).	Small sample size; Diagnostic accuracy of PET/MRI during the pre-operative period of endometrial cancer was not evaluated; Correlation of SUVmax and ADCmin to the parameters, including disease-free and overall survival, was not studied.

PET: Positron Emission Tomography; MRI: Magnetic Resonance Imaging; ADC: Apparent Diffusion Coefficient; SUV: Standardized Uptake Volume; CT: Computed Tomography; FDG: (18)F-fluorodeoxyglucose; DWI: Diffusion Weighted Imaging; PPV: Positive Predictive Value; NPV: Negative Predictive Value; PCI: Peritoneal Cancer Index.

**Table 6:** Characteristics of PET/MRI studies in recurrent gynecological malignancies.

Serial Number	Study	Year of Publication	Type of Study	Total Number of Patients in the Study	Objective of the Study	PET MR Machine Details	Results	Limitations
1	Sawicki et al. [96]	2017	Retrospective	71	To evaluate the impact of PET/MRI findings in the management of recurrent pelvic cancer, compared with MRI alone.	Biograph mMR PET/MRI scanner	PET/MRI is significantly superior in categorizing malignant lesions compared to MRI (99.2% vs. 79.3%; *p* < 0.001). It identified 100% of cancer recurrence compared to MRI, which identified only 83.6% of the recurrent tumors (*p* < 0.01).	Histopathological sampling was not performed; Heterogeneous patient population in terms of cancer types.
2	Grueneisen et al. [97]	2014	Prospective	34	To compare the diagnostic value of whole-body PET/MRI to whole-body MRI in the assessment of recurrent gynecological pelvic malignancies.	Biograph mMR PET/MRI scanner	In addition to superior lesion contrast and diagnostic confidence (*p* <0.001), PET/MRI identified 99% of malignant lesions compared to MRI alone, which discovered 89% of the malignant lesions.	Small sample size; Absence of standard criteria for the histological confirmation of the suspected malignant lesions.
3	Kirchner et al. [98]	2017	Retrospective	43	To study the diagnostic performance of ultrafast PET/MRI sequences (T2W, contrast-enhanced T1W, and SUV) compared to PET/CT and CT for the staging of recurrent pelvic cancers.	Biograph mMR PET/MRI scanner, Biograph mCT PET/CT	PET/MRI identified tumor recurrence equivalent to PET/CT. In addition, PET/MRI and PET/CT have equivalent diagnostic accuracies (94% vs. 92%), compared to the lower value of CT (53%).	Small patient cohort.
4	Grueneisen et al. [99]	2015	Retrospective	24	To compare the efficacy of fast PET/MRI and PET/CT in whole-body staging for recurrent pelvic malignancies.	Biograph mMR PET/MRI scanner	Although fast PET/MRI and PET/CT demonstrated equivalent diagnostic performance (86% vs. 84%), PET/MRI enables high-quality tumor restaging, though with a slightly prolonged scan duration.	Retrospective study; fast PET/MRI protocol is based on pre-obtained prolonged examination protocols; There is a time delay between the PET/MRI and PET/CT acquisitions that might have provided a gap for alteration in tumor metabolic activity.
5	Beirderwellen et al. [29]	2014	Retrospective	19	To study the diagnostic performance of PET/MRI and PET/CT in the evaluation of recurrent ovarian and cervical cancers.	Biograph mMR PET/MRI scanner; Biograph mCT 128 PET/CT scanner	Although the lesions were equivalently identified on PET/MRI and PET/CT (*p* > 0.05), the PET/MRI demonstrated higher diagnostic confidence in malignant (*p* < 0.01) and benign (*p* < 0.05) lesions.	Limited patient cohort; Modified reference standard was applied for the histological tumor classification.
6	Kitajima et al. [100]	2013	Retrospective	30	To study the accuracy of retrospectively fused PET and MRI images in the assessment of locoregional and nodal staging of endometrial cancer.	Discovery PET/CT 690, Signa Echo speed plus Excite MRI; Fused PET/MR images on advantage windows workstation	Fused PET/MRI is equivalent to MRI in regards to T-staging and PET/CT in regards to N-staging.	Retrospective study; PET/CT was not performed in all cases; Small sample size; Histopathological correlation was not possible for 53% of cases, as they did not undergo lymphadenectomy; Pelvic MR images instead of whole-body images were used for PET/MRI fusion; hence, distant metastases could not be studied.

PET: Positron Emission Tomography; MRI: Magnetic Resonance Imaging; ADC: Apparent Diffusion Coefficient; SUV: Standardized Uptake Volume; CT: Computed tomography; FDG: (18)F-fluorodeoxyglucose; DWI: Diffusion Weighted Imaging.

**Table 7:** Characteristics of PET/MRI studies in ovarian cancer.

Serial Number	Study	Year of Publication	Type of Study	Total Number of Patients in Study	Objective of Study	PET MR Machine Details	Results	Limitations
1	Tsuyoshi et al. [93]	2020	Retrospective	103	To evaluate the diagnostic value of PET/MRI in ovarian cancer.	Whole-body PET/MRI scanner	PET/MRI has superior sensitivity and specificity than ceCT and ceMRI on M-staging and has equivalent diagnostic potential to ceCt/ceMRI on T- and N-staging.	Retrospective study; Small sample size; Heterogeneous population; Histopathological correlation could not be performed in a few patients.

PET: Positron Emission Tomography; MRI: Magnetic Resonance Imaging; ceCT: Contrast-Enhanced Computed Tomography; ceMRI: Contrast-Enhanced Magnetic Resonance Imaging.

**Table 8:** PET/MRI Clinical Trials status.

Serial Number	ClinicalTrials.gov Identifier	Recruitment Status	Location (Sponsor/Collaborator/Country)	Study Design (Study Type/Actual Enrollment)	Official Title	Outcome Measure (Primary Outcome)	Outcome Measure (Secondary Outcome)
1	NCT03965481	Recruiting	M.D. Anderson Cancer Center, Houston/National Cancer Institute (NCI)/United States	Interventional/60 Participants	Comparing Accuracy of PET/MRI vs. ceCT in Assessment of Peritoneal Disease for resectability in Patients with Ovarian Cancer or Highly Suspected Ovarian Cancer	The accuracy of lesion detection will be summarized by modality using frequencies and percentages. The McNemar test will be utilized to compare the accuracies of PET/MRI and ceCT. Other diagnostic metrics (sensitivity, specificity, positive predictive value, and negative predictive value), and 95% confidence intervals will be estimated. The effect of patient and tumor characteristics on diagnostic precision will be evaluated using a logistic regression model.	Diagnostic accuracy by location will be analyzed using linear regression or generalized linear regression models where applicable. Where applicable, response status will be analyzed using linear regression or generalized linear regression models. Imaging and genomic data analysis correlation between imaging and genomic data will be analyzed using linear regression or generalized linear regression models where applicable.
2	NCT04454450	Active, not recruiting	Memorial Sloan Kettering Cancer Center, New York/Not provided/United States	Interventional/10 participants	Integration of Radiomic Analysis into the Multi-Modal Profiling of High-Grade Serous Ovarian Cancer	Radiologist-defined tumor volumes will be used to train and validate a machine-learning algorithm for generating segmentations. The data will be split into 70–30% training and test sets. The comparisons will be performed on a slice-by-slice and lesion-by-lesion basis, which should ensure sufficient examples for testing.	Not Provided
3	NCT03302156	Recruiting	University of Wisconsin, Madison/University of Wisconsin, Madison/United States	Interventional/52 participants	PSMA PET/MRI in Gynecological Cancers	Estimate the frequency with which PSMA PET and MR imaging and final IHC staining disagree in their classifications of the presence of disease.	Record the normal biodistribution of PSMA as detected in normal female controls by the resulting PET imaging. The radiodosimetry of PSMA-based 18F-DCFPyL will be measured in normal female controls via the resulting PET images. Record the distribution of PSMA in cancer tissue.
4	NCT04212910	Recruiting	IRCCS San Raffaele/IRCCS San Raffaele/Italy	Observational/101 participants	Stratifying Endometrial Cancer Patients Using a PET/MRI Prognostic Model	MRI evaluation with tumor volume measurement and correlation to surgical specimen, as well as changes in ADC normal versus tumor myometrium. Myometrial invasion depth. DCE-MRI perfusion parameters will distinguish between normal and tumor myometrium perfusion. Positive lymph node evaluation. Evaluation of PET imaging with PET-positive lymph nodes and SUV values for tumor/positive lymph node/metastases.	Measure tumor angiogenesis with angiogenesis marker CD1. Correlate PET- and MRI-derived functional and morphological parameters with histology using imaging-derived parameters correlated with histopathology.
5	NCT05483023	Not yet recruiting	University of North Carolina Lineberger Comprehensive Cancer Center/Radiological Society of North America/United States	Interventional/8 participants	18-FFNP PET/MRI as a Potential Biomarker of Response to Progesterone Therapy in CAH and Grade 1 Endometrial Cancer	Sensitivity of 18F-FFNP PET/MRI for predicting response to progestin therapy in CAH/EC patients and specificity of 18F-FFNP PET/MRI for predicting response to progestin therapy in CAH/Endometrial cancer patients.	First, to correlate FFNP Mean Standardized Uptake Value (SUVmean) at baseline and on repeat examination with estrogen and progesterone receptor expression in the CAH/Endometrial cancer tissues at baseline and after six months of treatment. Second, to correlate FFNP SUVmax at baseline and on repeat examination with estrogen and progesterone receptor expression in the CAH/Endometrial cancer tissues at baseline and after six months of treatment.
6	NCT02285192	Active, not recruiting	Memorial Sloan Kettering Cancer Center/Memorial Sloan Kettering Cancer Center/United States	Interventional/42 participants	Positron Lymphography Via Intracervical 18F-FDG Injection for Pre-surgical Lymphatic Mapping in Stage IB1 Cervical Cancer and High-grade Endometrial Cancer	The diagnostic accuracy of the Positron Lymphograph will be defined in terms of sensitivity and will consist of a pathology review of labeled, excised specimens compared with lymph node imaging data acquired preoperatively.	To evaluate several standard uptake values (SUV) (18F-FDG avidity), they will assess the ability of SUV to predict malignant disease. The continuous variable of SUV assigned to a given lymph node during Positron Lymphography will be compared with each labeled lymph node’s pathologic assessment (benign vs. malignant). The SUV assigned to a given lymph node is performed using imaging software and is not up to the radiologist’s discretion.
7	NCT05390021	Not yet recruiting	Massachusetts General Hospital/Massachusetts General Hospital/United States	Interventional/33 participants	Diagnostic Performance of PET/MRI Versus Standard of Care Imaging (PET/CT and/or CT and/or PET) in Preoperative Women With Presumed Early-stage High-Grade Endometrial Carcinoma	Percentage of patients with a metastatic lesion noted on PET/MRI that have a true malignancy noted on surgical pathology of that lesion (Sensitivity of PET/MRI to detect metastatic lesions). Percentage of patients without any metastatic lesions noted on PET/MRI that do not have malignancy noted on surgical pathology (Specificity of PET/MRI to detect an absence of metastatic lesions).	Not Provided
8	NCT05480995	Not yet recruiting	University of North Carolina, Chapel Hill/Eunice Kennedy Shriver National Institute of Child Health and Human Development (NICHD)/United States	Interventional/24 participants	Evaluation of Endometriosis With 18F-FFNPe PET/MRI	Sensitivity and Specificity of 18F-FFNP PET/MRI for evaluating endometriosis.	Diagnostic accuracy of PET/MRI. Correlation of uptake values (SUV-max) with EHP-30scale controlling for covariates. Correlation of uptake values (SUV-max) with pain level using a controlling for covariates.
9	NCT04347135	Enrolling by invitation	Annie (Annie) T. Packard, Mayo Clinic/Annie (Annie) T. Packard/United States	Interventional/10 participants	Pilot Study Evaluating Endometriosis With 16α-(18)F-fluoro-17β-estradiol ([F-18] FES) PET/MRI	Detection of Endometriosis: Comparisons will be made descriptively between conventional MRI imaging, FES PET/MRI imaging, and surgical/pathological findings. Outcome data include a number of detected lesions, differences in the accuracy of detection of active disease versus inactive fibrosis, and confidence score.	Not Provided
10	NCT04219904	Recruiting	M.D. Anderson Cancer Center/National Cancer Institute (NCI)/United States	Interventional/25 participants	Evaluation of Resectable Cervical Carcinoma With PET/MRI	Accuracy of diagnosing the depth of invasion on positron emission tomography/magnetic resonance imaging (PET/MRI).	Determine the relationship with pathology while assessing the lymph node involvement by PET/MRI. Inter-observer variability of PET/MRI. Quantitative imaging parameters of the tumor to correlate volumetric size, BOLD, ADC, IVIM analysis, DTI, DCE, MTV, TLG, standardized uptake value (SUV), and GMR with LVSI and tumor grade on surgical pathology which serves as the gold standard.
11	NCT01899404	Active, not recruiting	University Health Network, Toronto/Princess Margaret Hospital, Canada/Canada	Interventional/25 participants	A Pilot Prospective Study of the Utility of DCE-MRI, Diffusion Weighted MRI (DWI), and Positron Emission Tomography (PET) Imaging With 18F-Fluorodeoxyglucose (18FDG) in Brachytherapy for Cervix Cancer	Target delineation in brachytherapy using standard T2-weighted MRI versus DCE-MRI, DWI, and FDG-PET/CT imaging: gross tumor volume and high-risk clinical target volume in patients with cervical cancer. Target delineation using standard MRI acquired during the last week of EBRT fused to planning CT versus full MRI-guided brachytherapy.	Follow-up imaging (MRI and 18FDG PET) versus the imaging done at the time of brachytherapy. Imaging techniques for visualizing the brachytherapy applicator.
12	NCT05355558	Recruiting	National Cancer Center, Singapore/National Cancer Center, Singapore/Singapore	Interventional/15 participants	Novel Functional Imaging Technique With FLT-PET/MRI For Staging, Response Assessment, and Radiation Treatment Planning in Cervix Cancer	To determine the feasibility of [18F]FLT-PET/MRI imaging for early prediction of treatment by comparison of changes in baseline SUV uptake at week 4–5 of External Beam Radiotherapy.	Compare SUV uptake of FLT with FDG PET at diagnosis. Compare SUV uptake of FLT before and after chemoradiotherapy. To compare differences in tumor and regional staging between PET/MRI, PET/CT, and MRI scans, determined from the tumor size and extent of local involvement. Assess the feasibility of PET/MRI in the radiation treatment planning workflow with respect to the adequacy of image quality and image fusion of PET/MRI data with the treatment planning CT for marrow-sparing RT plan. Compare changes in stimulated radiation treatment volume derived from PET/MRI vs. PET/CT vs. MRI. Compare VMAT versus IMRT versus proton versus tomotherapy for the best marrow-sparing plan. To determine the correlation of [18F]FLT parameters as a baseline during treatment and change in [18F] FLT parameters with clinical outcome and response.

PSMA: Prostate Specific Membrane Antigen; IHC: Immunohistochemistry; 18F-DCFPyL: 18-fluorofuranylnorprogesterone (FFNP); PET: Positron Emission Tomography; MRI: Magnetic Resonance Imaging; ADC: Apparent Diffusion Coefficient; SUV: Standardized Uptake Volume; CT: Computed tomography; FDG: (18)F-fluorodeoxyglucose; 18F-FLT: 3’-deoxy-3’-^18^F-fluorothymidine; DCE-MRI: Dynamic Contrast-Enhanced MRI; DWI: Diffusion Weighted Imaging; BOLD: Blood Oxygen Level-Dependent; DTI: Diffusion Tensor Imaging; DCE: Dynamic Contrast-Enhanced; MTV: Metabolic Tumor Volume; TLG: Total Lesion Glycolysis; GMR = Gradient motion rephrasing; EBRT = External beam radiation therapy; RT = Radiotherapy; VMAT = Volumetric modulated arc therapy; IMRT = Intensity- modulated radiation therapy; IVIM: Intravoxel Incoherent Motion; Complex Atypical Hyperplasia (CAH); Endometriosis Health Profile (EHP-30); visual analog scale (VAS).

**Table 9:** Characteristics of PET/MRI meta-analysis.

Serial Number	Study	Year of Publication	Type of Study	Total Patients in Study	Objective	Result	Limitation
1	Virarkar et al. [105]	2020	Systematic review and meta-analysis		Meta-analysis of studies performed on PET/MRI in gynecologic cancers.	Patient-based analysis: Sensitivity, specificity, diagnostic odds ratio, and AUC of PET/MRI were 74%, 90%, 26, 0.834, respectively; While the respective values were 88%, 88%, 50, and 0.922, respectively, on lesion-based analyses.	The studies that were included were heterogeneous; Publication bias could not be ruled out entirely; Lack of standard guidelines, scanning protocols, and timing of PET/MRI among the included studies.
2	Nie et al. [106]	2017	Systematic review and meta-analysis		Meta-analysis of studies performed on PET/MRI in gynecologic cancers.	Patient-based analysis: Sensitivity and specificity of PET/MRI were 95% and 95%, respectively, while the values for lesion-based analyses were 89% and 87%, respectively.	Heterogeneity of the studies; Publication-biased tests were not performed; Lower number of studies were included.
3	Nguyen et al. [1]	2020	Systematic review and meta-analysis		Systematic review of studies on the role of PET/MRI in gynecological malignancies.	PET/MRI has comparable efficacy to PET/CT in the staging and restaging of gynecologic tumors; Mild-to-moderate inverse correlation was observed between SUV and ADC values which may predict the risk stratification and grading.	
4	Virarkar et al. [107]	2020	Systemic review and meta-analysis		Meta-analyses of the studies to compare the diagnostic performance of PET/CT and PET/MRI in gynecological malignancies.	Patient-based analysis: Sensitivity, specificity of PET/CT were 63% and 92%, while that of PET/MRI were 73% and 92%, respectively. Lesion-based analysis: Sensitivity and specificity of PET/CT were 82% and 87%, while that of PET/MRI were 85% and 89%, respectively.	Small cohort of studies; High heterogeneity; Publication biases could not be excluded completely; Spectrum bias due to non-standardized protocols in the included studies.
5	Zheng et al. [103]	2018	Systemic review and meta-analysis		Meta-analyses of studies to describe the role of PET/MRI in recurrent female pelvic malignancies; malignancies of female patients using a meta-analysis.	Patient-based analysis: Sensitivity and specificity of PET/MRI were 96% and 95%, respectively, while the respective values based on lesion analysis were 99% and 94%, respectively.	Small cohort of studies; Unpublished studies were not included; 57% of the included studies were retrospective.

PET: Positron Emission Tomography; MRI: Magnetic Resonance Imaging; ADC: Apparent Diffusion Coefficient; SUV: Standardized Uptake Volume; CT: Computed tomography; FDG: (18)F-fluorodeoxyglucose; DWI: Diffusion Weighted Imaging; AUC: Area Under the Curve.
